# Metabolic inhibition of meloxicam by specific CYP2C9 inhibitors in *Cunninghamella blakesleeana* NCIM 687: in silico and in vitro studies

**DOI:** 10.1186/s40064-016-1794-4

**Published:** 2016-02-24

**Authors:** G. Shyam Prasad, K. Srisailam, R. B. Sashidhar

**Affiliations:** Department of Biochemistry, University College of Science, Osmania University, Hyderabad, Telangana State India; Department of Pharmacy, University College of Pharmaceutical Sciences, Satavahana University, Karimnagar, Telangana State India

**Keywords:** CYP2C9 inhibitors, *Cunninghamella blakesleeana*, Drug interaction, In silico studies, LC–MS, Meloxicam

## Abstract

Specific inhibitors of Cytochrome P4502C9 enzyme (CYP2C9) viz. clopidogrel, fenofibrate fluvoxamine and sertraline at concentration of 50, 100, 150 and 200 µM were employed to investigate the nature of enzyme involved in bioconversion of meloxicam to its main metabolite 5-OH methyl meloxicam by *Cunninghamella blakesleeana*. Virtual screening for interaction of specific CYP2C9 inhibitors with human CYP2C9 enzyme was performed by molecular docking using Auto dock vina 4.2 version. The in silico studies were further substantiated by in vitro studies, which indicated fenofibrate to be a potent inhibitor of CYP2C9 enzyme followed by sertraline, clopidogrel and fluvoxamine, respectively. Two-stage fermentation protocol was followed to study metabolism of meloxicam and its inhibition by different CYP2C9 inhibitors. Meloxicam metabolites were identified using HPLC, LC–MS analysis and based on previous reports, as 5-OH methyl meloxicam (M1), 5-carboxy meloxicam (M2) and an unidentified metabolite (M3). All the inhibitors tested in the study showed a clear concentration dependent inhibition of meloxicam metabolism. The results suggest that the enzymes involved in metabolism of meloxicam in *C. blakesleeana* are akin to mammalian metabolism. Hence, *C. blakesleeana* can be used as a model organism in studying drug interactions and also in predicting mammalian drug metabolism.

## Background

Drug–drug interaction is a condition where one drug affects the activity of the other by increasing or decreasing its activity. These interactions are seen in patients suffering from chronic ailments such as congestive heart failure, rheumatic diseases, hypertension, cancer, and human immunodeficiency which require multiple drug therapy and may result in adverse drug interactions manifested as a loss in drug efficacy (Doucet et al. 2002). Co-administration of ketoconazole and terfenadine was reported to cause potentially life-threatening ventricular arrhythmias (Manahan et al. 1990) and an interaction between sorivudine and fluorouracil also resulted in fatal toxicity (Watabe 1996; Sokuda et al. 1997). Cytochrome enzymes play a major role in metabolizing drugs and the activity of this group of enzymes or a single CYP can determine patient’s response to drug therapy. Therefore, modulation of the activity of CYPs by a given drug is a critical issue for the assessing the safety and efficacy of a drug. Inhibition of CYP can increase systemic exposure leading to severe toxic side effects of the drug or another concomitantly given medication that is metabolized by the respective CYP(s) (Romet et al. 1994; Wandel et al. 1998). Cardiac toxicity caused by co-administering the antihistamine terfenadine with the antifungal ketoconazole or the antibiotic erythromycin is an example whereby inhibition of CYP 3A4 results in elevated terfenadine levels, resulting in prolongation of the QTc interval (Venkatakrishna et al. 2000). Similarly, the increased bleeding in patients on warfarin therapy has been attributed to inhibition of its metabolism (Chan 1998).

Progress in CYP enzymology and biochemistry in the recent past suggests that, the drug interactions are based on enzyme inhibition. This aspect can be further investigated using in vitro techniques with microsomes, expressed enzymes, or cell systems (Pichard et al. 1990; Von Moltke et al. 1994). An understanding of the drug-metabolizing enzymes role in the clearance of compounds and of drug–drug interactions caused by co-administered medications is an essential component of both the drug discovery process and its therapeutics. Currently variety of tools are available to assess the potential of a drug in inhibiting different P450 enzymes invitro which include human liver tissue, cDNA expressed P450 enzymes, and specific probe substrates. Among them, human liver microsomal preparations are method of choice. The utility of tissues from individual donors for inhibition of enzyme selective studies is limited by sufficiency of catalytic activity present in the tissue. Alternately, when a specific enzyme is to be investigated, recombinant P450s can be used. The most preferred test systems are microsomes and recombinant p450 enzymes as they are readily available than human hepatocytes and p450 kinetic measurements are not confounded with other metabolic processes or cellular uptake. A major disadvantage of microsomes or recombinant enzymes is that they do not represent the true physiological environment. (e.g., not all Phase II enzymes are present) if that is of interest to study. These processes are also associated with high cost.

Microorganisms and their enzymes have proved to be versatile biocatalysts and are involved in the transformation of complex organic materials to produce bioactive compounds and some of them have the potential to metabolise or degrade the desired compounds. This process is simple, hazard free, minimizes the problems of racemization, isomerization, epimerization and rearrangement that generally occur during chemical process, renewable or recycled many times, economical and ecofriendly. Most of the fungal cultures were employed in biotransformation of drugs especially the fungus belonging to the genus *Cunninghamella* is well documented, also as a model of mammalian biotransformation (Zhang et al. 2006; Lisowska et al. 2006). The advantages of microbial systems as an in vitro model for drug biotransformation include its low cost, ease of handling, scale up capacity and its potential to reduce the use of animals (Pupo et al. 2007). Further it is an efficient and environmentally friendly process.

Meloxicam, the anti-inflammatory drug used for the treatment of rheumatic disease is mainly metabolized to 5-hydroxymethyl metabolite that is further converted to a 5-carboxy metabolite (Schmid et al. 1995) and to other derivatives. The 5-hydroxylation of meloxicam is predominantly catalyzed by CYP2C9 and with a minor contribution of CYP3A4 (Chesne et al. 1998). Any medication inhibiting CYP2C9 can potentially block the conversion of meloxicam into its metabolites. 5-OH methyl meloxicam was found to be the major metabolite in mammals and the microbes studied so far (Busch et al. 1998; Prasad et al. 2009a).

In our earlier studies, three major metabolites of meloxicam viz. 5-OH methyl meloxicam (M1), 5-carboxy meloxicam (M2) and an unidentified metabolite (M3) were recorded employing *C. blakesleeana* as a model organism (Prasad et al. 1998). In the present investigation, we report the inhibition of the enzyme involved in the bioconversion of meloxicam to 5-OH methyl meloxicam using specific CYP2C9 inhibitors viz. clopidogrel, fenofibrate, fluoxamine and sertraline in *C. blakesleeana.*

## Results and discussion

### In silico studies

The CYP2C9 (receptor) 3D structure with PDB I.D 1OG2 was obtained from PDB data base and the ligands from PubChem and are converted to 3D PDB files. After preparing receptor (CYP2C9) and 4 ligands (clopidogrel, fenofibrate, sertraline and fluvoxamine), each ligand individually was subjected to docking with human cytochrome P450 (CYP) 2C9 enzyme (receptor) and the docking was found to be successful based on the evident formation of complexes of CYP2C9 enzyme with specific human CYP2C9 enzyme inhibitors. The hydrogen bond interactions, binding energy, bond length, RMSD, active site residues and orientation of the docked compound within the active site were visualized using PyMOL software. All the test compounds screened showed best fit RMSD value of 0.000, indicating statistically significant interaction. The binding energies were found to be, −7.8, −7.3, −6.9 and −6.2 kcal mol^−1^ for fenofibrate, sertraline, clopidogrel, and fluvoxamine, respectively (Table 1). The negative and low value of ΔG indicated a strong favorable bonding between CYP2C9 enzyme and the ligands, suggesting that the bound ligand was in its most favorable conformations.

**Table 1:** Ligands docked to crystal structure of human cytochrome P450 CYP2C9 (PDB: Code 10G2)

Ligand name	Binding energy (kcal/mol)	Number of hydrogen bonds	Distance (Å)	Residues involved in hydrogen bond	Atom of compound
Clopidogrel	−6.9	1	2.6	ARG-97/2HH1	O-20
Fenofibrate	−7.8	1	2.4	ASN-217/1HD2	O-24
Fluvoxamine	−6.2	2	2.5 2.4	GLN-214/1HE2 ASN-217/1HD2	O-21
Sertraline	−7.3	Hydrophobic interactions	**–**	**–**	**–**

Fenofibrate showed binding energy of −7.8 which was found to be higher as compared to other inhibitors tested with one hydrogen bond interacting with CYP2C9 enzyme in the complex at O-24 position of the ligand. The residue involved in this interaction was ASN-217. Sertraline was found to be potent inhibitor of CYP2C9 next only to fenofibrate with binding energy of 7.3 kcal/mol. Interestingly, sertraline could not form any hydrogen bonding with receptor (CYP2C9) indicating the interaction to be hydrophobic. The surrounding amino acids Arg-433, Thr-301, Val-113 may be involved in forming hydrophobic interactions with sertraline.

Clopidogrel showed binding energy of −6.9 kcal/mol, forming one hydrogen bond with the receptor and was less potent as compared to fenofibrate and sertraline. The residue involved in forming hydrogen bonding was identified as ARG-97. Further, clopidogrel was involved in forming hydrogen bonding with oxygen at position O-20.

Even though, fluvoxamine showed two hydrogen bonds with the receptor with a bonding energy of −6.2, which was least potent as compared to other inhibitors tested. GLN-214 and ASN-217 are the two aminoacid residues that are involved in forming hydrogen bonds. The oxygen (O-21) of the ligand was involved in the bonding.

**Fig. 1:**
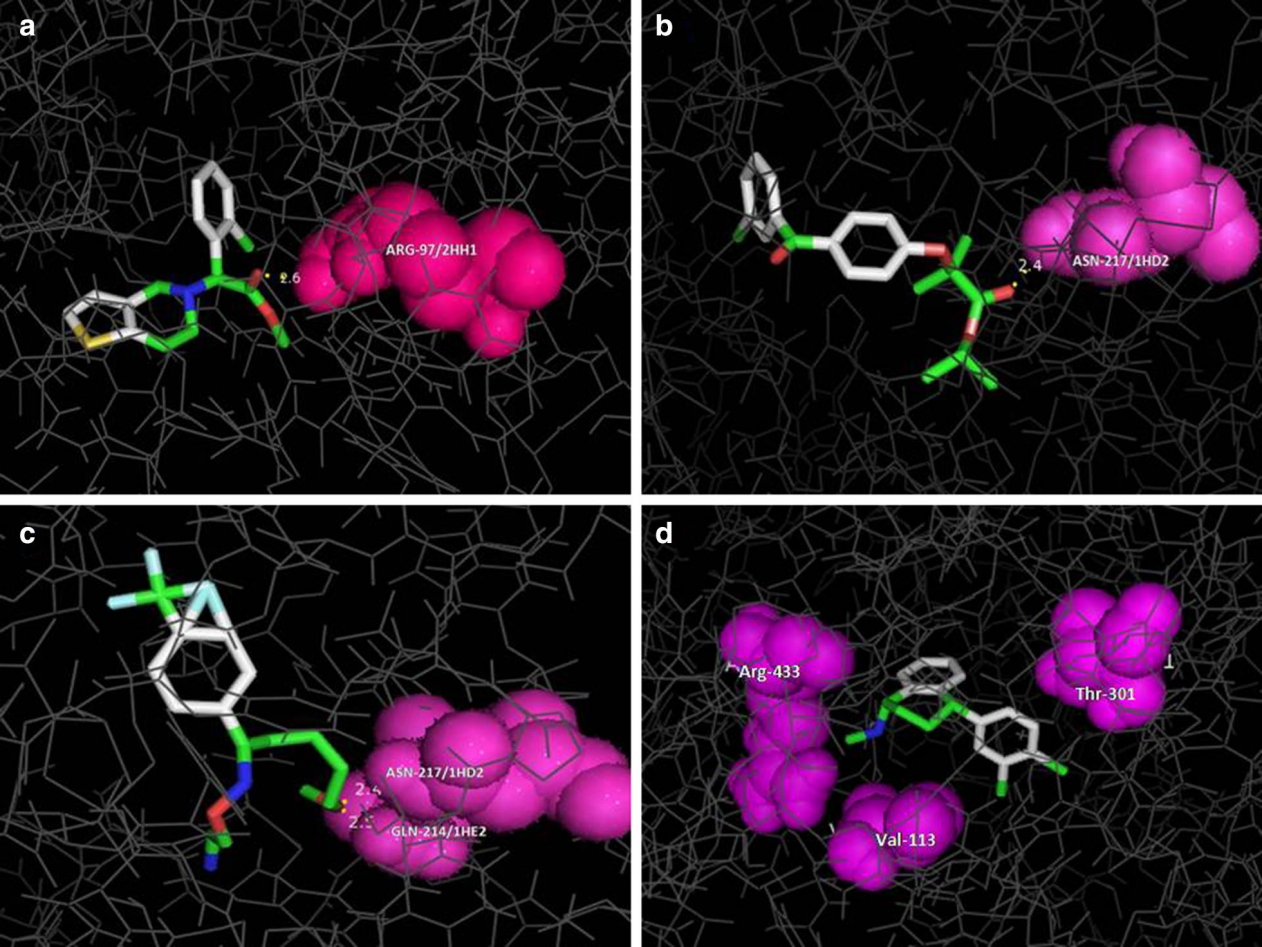
Virtual screening for interaction of specific CYP29 inhibitors (**a**–**d**) clopidogrel, Fenofibrate, fluvoxamine, sertraline with human CYP2C9 (PDB code: 1OG2) enzyme by molecular docking

The details of binding energies, number of hydrogen bonds formed and catalytic site residues involved in protein–ligand complex of CYP2C9 with different ligands are depicted in Table 1 and Fig. 1.

### In vitro studies

**Fig. 2:**
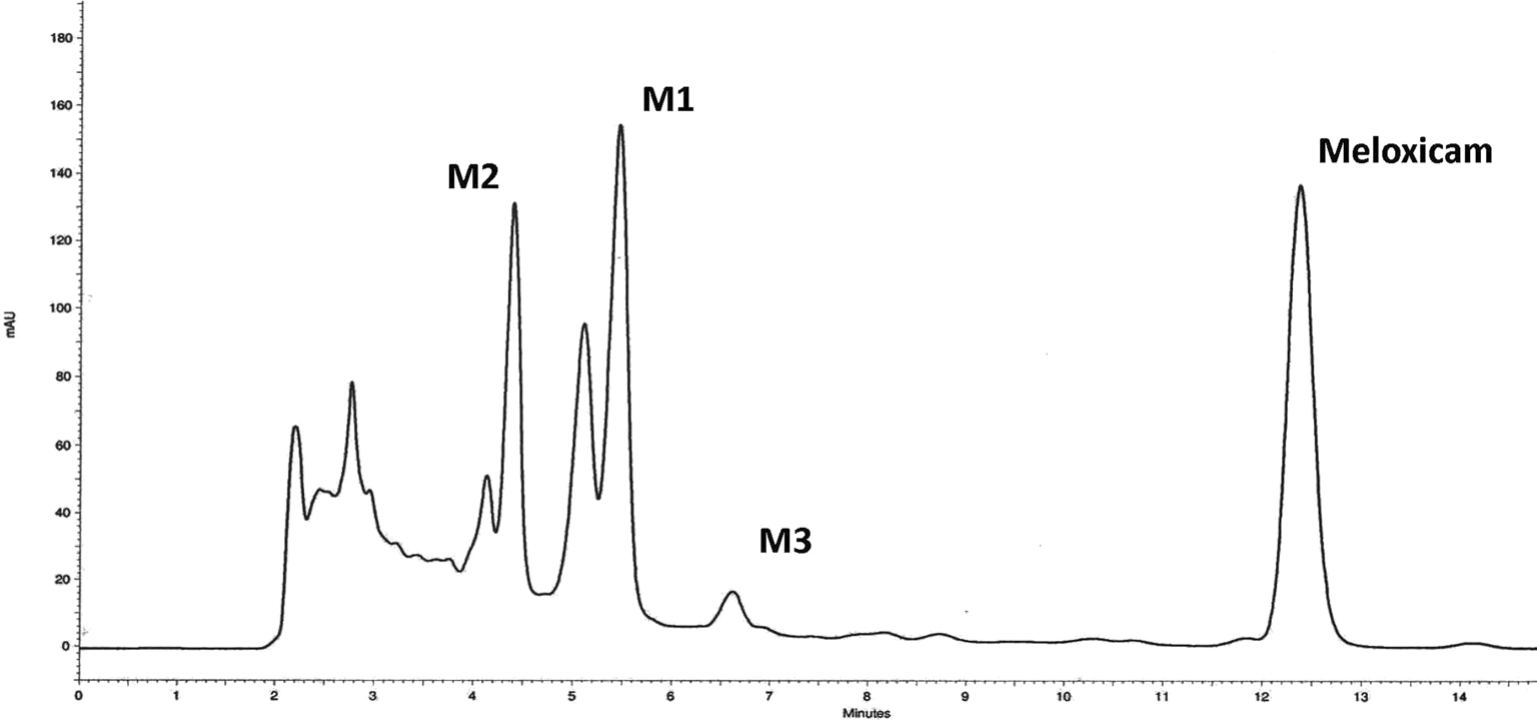
HPLC chromatogram showing metabolites of meloxicam in culture broth of *C. blakesleeana*

**Fig. 3:**
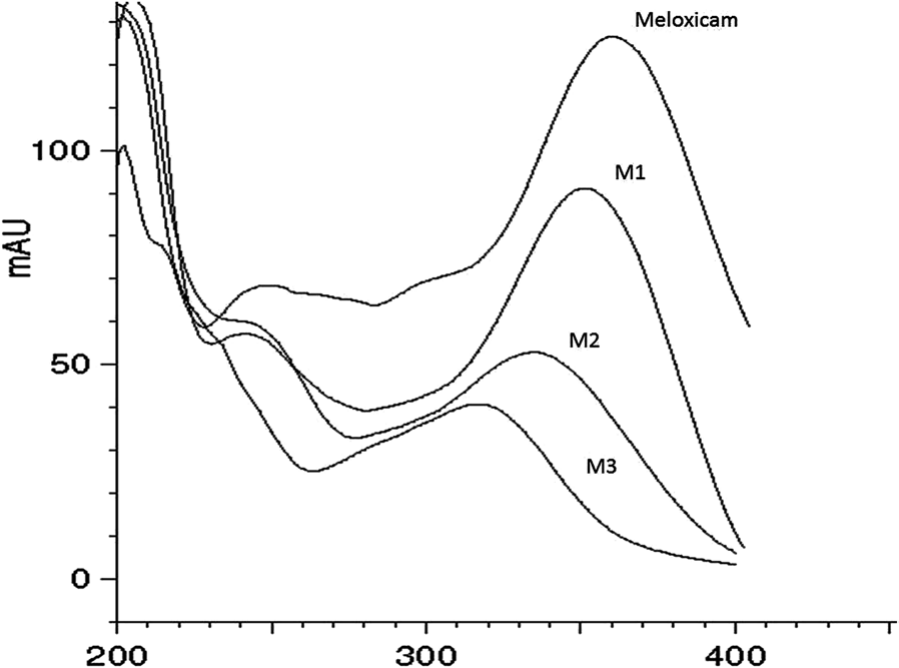
UV spectra of meloxicam and its metabolites produced by *C. blakesleeana* NCIM 687 using Photodiode array detector (PDA) of HPLC

In the present investigation, three metabolites of meloxicam M1, M2 and M3 employing *C. blakesleeana* were identified as reported earlier (Prasad et al. 2009a) evidenced from HPLC analysis of ethyl acetate extract of the test and control samples. The metabolite peaks were identified in HPLC analysis of test sample basing on similarity in UV spectra using photodiode array detection. The chromatogram of culture control (fungus without drug) showed no metabolites peaks and substrate control (drug without fungus) showed the presence of meloxicam only. The retention time for the metabolites M1, M2 and M3 were observed to be 5.5, 4.4, 6.6 min, respectively; while the retention time of meloxicam was found to be at 12.4 min in HPLC analysis (Fig. 2). The UV spectra of meloxicam and its metabolites were found to be similar indicating the parent molecule and its biotransformed metabolites had similar UV absorption pattern (Fig. 3). This indicates that meloxicam has undergone minor structural changes while basic moiety remains intact. The metabolites were quantified based on area under the peak recorded in the HPLC analysis taking the drug and metabolites peak areas together as 100 %.

**Fig. 4:**
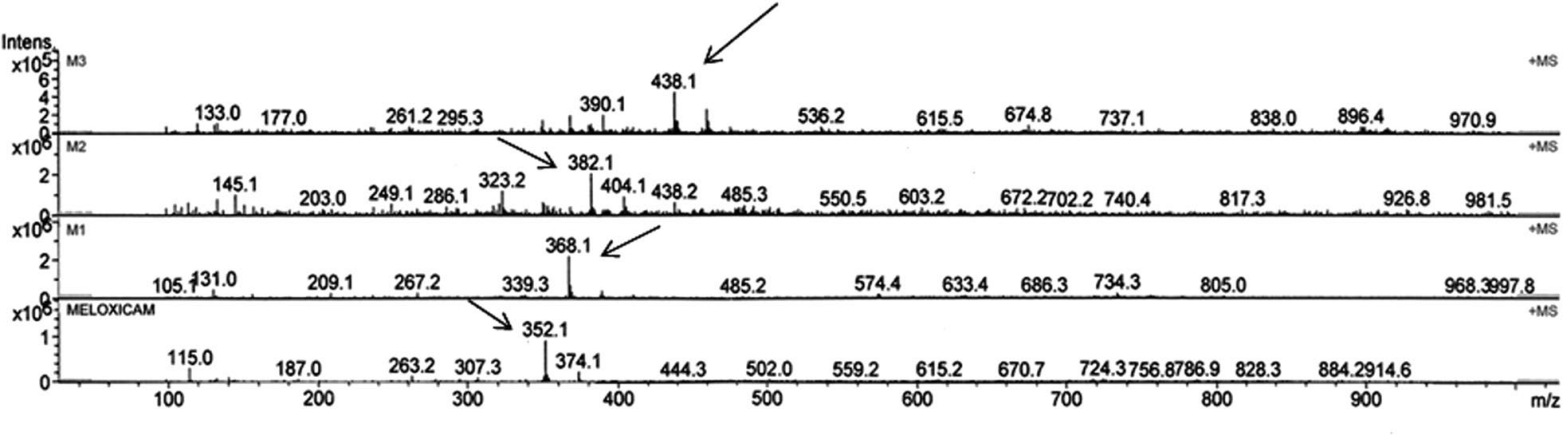
LC-MS spectra of metabolites detected in meloxicam fed culture broth of *C. blakesleeana*

**Fig. 5:**
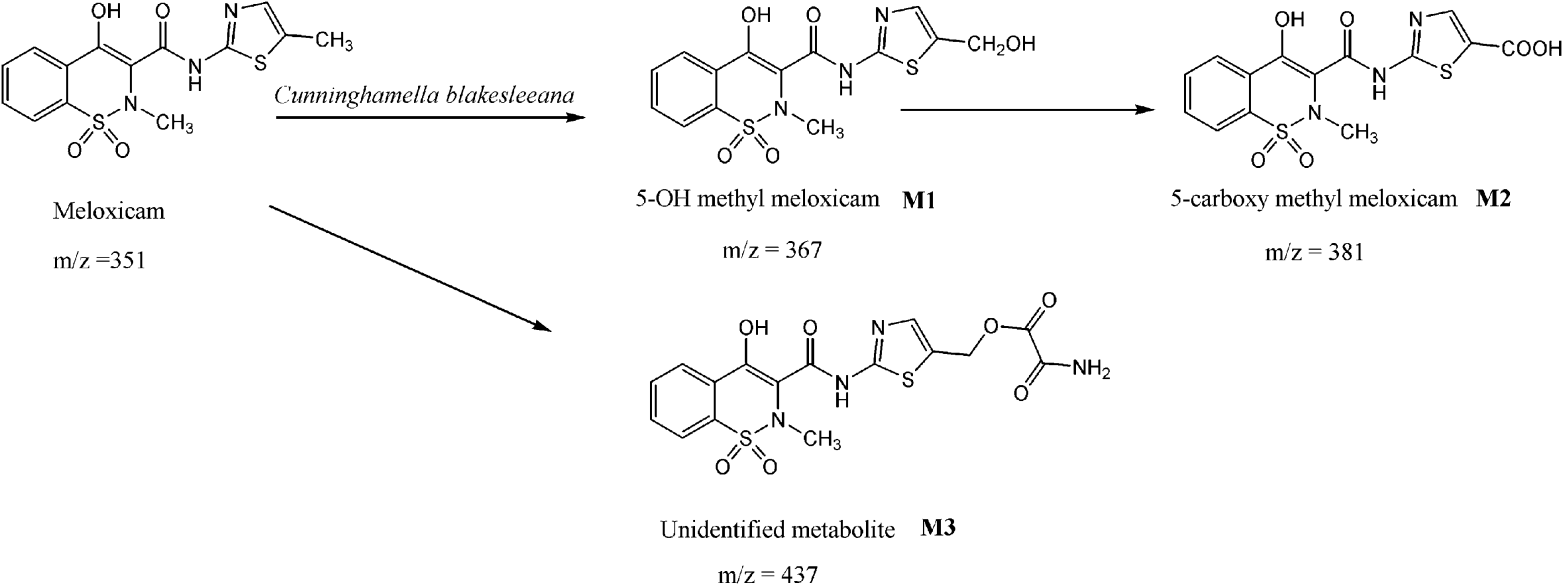
Metabolic pathway of meloxicam in *C. blakesleeana* NCIM 687

The structure elucidation of the metabolites was carried out from the *m*/*z* values of the protonated molecular ion peaks obtained in LC–MS analysis (Fig. 4), HPLC retention times, chromatographic elution order and with earlier reports (Busch et al. 1998; Prasad et al. 2009a).The metabolic pathway of meloxicam in *C. blakesleeana* was shown in Fig. 5.

LC–MS analysis of test sample showed a molecular ion at *m*/*z* = 368 [M + H] +, an increase of 16 *m*/*z* units to meloxicam indicating addition of a single oxygen atom. This suggests that the compound might be 5-OH methyl meloxicam (M1). The metabolite M1 formation from meloxicam was reported to mediate by cytochrome P450 2C9 with minor contribution of CYP3A4 enzyme in mammals (Chesne et al. 1998). This derivative of meloxicam was also reported in *Cunninghamella elegans,* horses (Aberg et al. 2009) and in *C. blakesleeana* NCIM 687 (Prasad et al. 2009a).

Metabolite M2 showed a molecular ion at *m*/*z* = 382 [M + H]^+^ an increase of 14 *m*/*z* units, indicating clearly further addition of an oxygen and removal of two hydrogen atoms from M1. This compound might be 5-carboxy meloxicam (M2). The production of metabolite M1 using *C. elegans* NCIM 690, *Saccharomyces cerevisiae* NCIM 3090, *Bacillus subtilis* MTCC 441, *Pseudomonas putida* NCIM 2783 and the production of both metabolites M1 and M2 using *Aspergillus niger* NCIM 589, *A. ochraceous* NCIM 1140, *C.echinulata* NCIM 691 was reported earlier (Prasad et al. 2009a, b) and by Aberg et al. (2009) in *C. elegans* and horses. These metabolites viz. M1, M2 were also detected in mammals showing similar metabolism of meloxicam (Busch et al. 1998; Aberg et al. 2009). The metabolites of meloxicam both M1 and M2 were reported to be pharmacologically inactive (Davies and Skjodt 1999).

A third metabolite with *m*/*z* 438 [M + H] + was recorded with 86 *m*/*z* units higher to meloxicam. The production of M3 using *C. blakesleeana* was also recorded by Prasad et al. (2009b). This is an unidentified metabolite of meloxicam (M3). A probable molecular structure of metabolite M3 was presented in Fig. 3. However, further investigations are needed to confirm its structure. The LC–MS data, of the metabolites are presented in Table 2.

**Table 2:** LC–MS data of meloxicam metabolites by *C. blakesleeana* NCIM 687

Metabolite	Rt	[M + H]+	Predicted molecular formula	Predicted reaction
Meloxicam	12.4	352	C_14_H_13_N_3_O_4_S_2_	**–**
5-OH methyl meloxicam (Ml)	5.5	368	C_14_H_13_N_3_O_5_S_2_	Hydroxylation
5-Carboxy meloxicam (M2)	4.4	382	C_14_H_11_N_3_O_6_S_2_	Carboxylation
Unidentified metabolite (M3)	6.6	438	–	–

To find the nature of enzyme involved in M1 production by *C. blakesleeana,* Specific inhibitors of CYP2C9 were used in the study and successful inhibitory action of tested inhibitors was observed.

Inhibition of 5-OH methyl meloxicam (M1) formation from meloxicam was studied at 50, 100, 150 and 200 µM inhibitor concentrations using known human CYP2C9 specific inhibitors viz. clopidogrel, fenofibrate, fluvoxamine and sertraline. Fungal culture with meloxicam without inhibitor was used as control to know the actual percent production of M1; The experimental results are presented in Table 3.

**Table 3:** Effect of different CYP2C9 inhibitors on meloxicam metabolism by *C. blakesleeana*

Control (without inhibitor)	Concentration of inhibitor	% of Ml produced + SD	#Dry weight of fungal biomass in mg
	–	75 ± 0.34	11.63 ± 0.04
Specific CYP2C9 inhibitors			
Clopidogrel (μM)	50	33.55 ± 0.74*	11.52 ± 0.02
	100	23.22 ± 0.24*	11.56 ± 0.03
	150	14.59 ± 0.91*	11.42 ± 0.02
	200	13.03 ± 1.96*	10.33 ± 0.02
Fenofibrate (μM)	50	39.13 ± 0.78*	11.62 ± 0.26
	100	36.45 ± 0.64*	11.52 ± 0.03
	150	25.36 ± 0.14*	11.54 ± 0.03
	200	7.89 ± 0.12*	11.51 ± 0.03
Fluvoxamine (μM)	50	35.48 ± 1.20*	11.74 ± 0.02
	100	33.89 ± 0.24*	11.61 ± 0.03
	150	27.91 ± 1.03*	11.57 ± 0.04
	200	22.53 ± 0.66*	10.31 ± 0.02
Sertraline (μM)	50	40.77 ± 0.50*	11.53 ± 0.005
	100	35.73 ± 0.95*	11.51 ± 0.02
	150	26.96 ± 0.72*	11.71 ± 0.02
	200	11.23 ± 0.44*	10.61 ± 0.03

The values are based on three independent experiments; values are Mean ± standard deviation

* P < 0.01; ^ #^ P < 0.05-non significant

From Table 3 it is clear that the yield of metabolite M1 was 75 % without addition of any inhibitors, while all the inhibitors employed in the present investigation indicated a clear concentration dependent inhibition of M1 formation from meloxicam, compared to the control.

At a concentration of 50,100 and 150 µM fenofibrate, sertraline and fluvoxamine showed similar  % inhibition of M1, while at 200 µM concentration, fenofibrate, sertraline and fluvoxamine resulted in 7.89, 11.23 and 22.53 % inhibition of M1, respectively. Among all the inhibitors tested, clopidogrel was found to achieve maximum inhibition of M1 formation at 100 and 150 µM concentrations, which may be attributed to the saturation of binding sites.

Biomass production in control and test samples of fungal cultures showed similar pattern of growth. There was no statistically significant difference (P < 0.05) between fungal growth in controls and experimental groups, wherein the added test molecules had not impacted the growth of the fungus (Table 3).

Fenofibrate at 50–200 µM and clopidogrel at 50–150 µM were found to be the potent inhibitors of M1 formation followed by sertraline and fluoxamine at 50–200 µM. Similar results with clopidogrel and fenofibrate was reported in human liver microsomes by Richter et al. (2004) and Vecera et al. (2011). Schmider et al. (1997) also reported inhibition of CYP2C9 using sertraline in human liver microsomes. Strong inhibition of CYP2C9 with fluvoxamine in human liver microsomes was also recorded by Hemeryck et al. (1999) and Madsen et al. (2011). Zing et al. (2010) also studied the influence of fluvoxamine on CYP2C9*3 and CYP2C9*13 enzymes by expressing in yeast cells.

In the present study, in silico analysis, was further substantiated by in vitro experimental data suggesting the inhibitory action of tested CYP2C9 inhibitors on CYP2C9 enzyme. Among inhibitors tested fenofibrate was found to be a potent inhibitor of M1 formation followed by sertralinee, fluoxamine and clopidogrel. Additionally, it is clear that CYP2C9 like enzyme exists in *C. blakesleeana* and had catalyzed the formation of 5-OH methyl meloxicam (M1) from meloxicam which, is akin to mammalian xenobiotics.

## Conclusion

In the present investigation, in silico studies clearly predicted the interaction of specific CYP2C9 inhibitors with human CYP2C9 enzyme. The drug docking studies were substantiated by in vitro studies. The metabolite M1 formation from meloxicam by *C. blakesleeana* was strongly inhibited using specific inhibitors of CYP2C9 viz. fenofibrate followed by sertralinee, clopidogrel and fluvoxamine. Similarly, metabolites of meloxicam reported in mammals were also detected employing *C. blakesleeana* as a model organism. This is indicative of the existence of a similar type of enzyme system present in fungus that is akin to mammals. Hence, *C. blakesleeana* may be used as a model organism in predicting drug–drug interactions as well as in studying mammalian drug metabolism, which provides true physiological environment as in animals.

## Methods

### Chemicals

Meloxicam was gifted by Unichem Laboratories Mumbai, India. Clopidogrel, fenofibrate fluvoxamine and sertraline were obtained from the Dr. Reddy Labs, Hyderabad, KU Warangal. Methanol was of HPLC grade obtained from Ranbaxy Laboratories Ltd., New Delhi, India. Peptone, yeast extract, NaCl, glucose, K_2_HPO_4_ and all other chemicals were obtained from Himedia Labs, Mumbai, India.

### Microorganism

*Cunninghamella blakesleeana* NCIM 687 was procured from National Chemical Laboratory, Pune, India. Stock cultures were maintained on Potato dextrose agar slants at 4 °C and sub cultured for every 3 months for maintaining viability.

### In silico studies: Virtual screening for interaction of specific CYP29 inhibitors with human CYP2C9 enzyme by molecular docking

In the present In silico study, specific human CYP2C9 enzyme inhibitors viz. clopidogrel, fenofibrate fluvoxamine and sertraline were used as ligands and were screened against the crystal structure of human cytochrome P450 CYP2C9 enzyme with PDB code 1OG2. An advanced molecular docking program Auto Dock Vina, version 4.2 available from http://vina.scripps.edu/ was applied to select ligand active against CYP2C9 enzyme and estimating the binding affinities (kcal mol^−1^). The CYP2C9 enzyme was downloaded from protein data bank (PDB code: 1OG2) and all the ligands were downloaded from PubChem database and optimised by removing water molecules and adding hydrogens. Later, both ligands and receptor was saved as pdbqt format. A grid box was adjusted to have center x = 14.846, y = 69.693, and z = 21.831 to cover the pocket with the main residues of enzyme binding site. The best conformation was chosen with the lowest docked energy, based on complete docking search (ten runs). The interactions of CYP2C9 enzyme with the ligands, hydrogen bonds, bond lengths, Root Mean Square Difference (RMSD) were analyzed using PyMOL software.

### In vitro studies: fermentation conditions

Microbial fermentation was carried out in a liquid broth containing (per liter) Yeast extract (5 g), glucose(20 g), peptone (5 g), NaCl (5 g), K_2_HPO_4_ (5 g). Specified quantities of the media ingredients were dissolved in distilled water, pH was adjusted to 6.0 with either 0.1 N HCl or 0.1 N NaOH and sterilized by autoclaving and cooled before inoculation. Transformation of meloxicam was performed by incubating culture flasks on a rotary shaker at 120 rpm and 27 °C according to two stage fermentation procedure. In the first stage, fermentation was initiated by inoculating a 100 ml culture flask consisting of 20 ml of liquid broth with loopful of spores obtained from freshly grown agar slant. After incubation for 48 h second stage cultures were initiated in the same medium using inoculum of 1.0 ml from the first stage culture per 20 ml of medium in a 100-ml culture flask and incubated for 24 h under similar conditions. Two mg of meloxicam (in 200 µl DMSO) was added to the culture flasks and incubated further for another 4 days.

Two types of controls were run simultaneously with the fermentation and worked up with the same method. One was culture control consisted of a fermentation blank in which the microorganism was grown under identical conditions without the substrate. The other was substrate control comprised of meloxicam added to the sterile medium without fungus and incubated under similar conditions. Three independent experiments were carried out.

### Extraction of metabolites

At the end of incubation period, the fungal mat was separated from the culture broth and dried, pH of the broth was acidified to 3.0 with orthophosphoric acid and the later was extracted with three volumes of ethyl acetate, the combined organic extracts were evaporated using a rotary vacuum evaporator and dried over a bed of sodium sulfate. The resultant residues were analyzed by HPLC and LC–MS for presence and identification of metabolites.

### Analytical procedures

HPLC analysis was performed according to the method described by Prasad et al. (2009a). The samples were analyzed using an Agilent 1260 infinity system (USA) by injecting 20 µl into the syringe-loading sample injector (Agilent, Model G1328C). The column used was Agilent, Zorbax Eclipse XDB-C_18_, 4.6 × 250 mm and 5 µm (USA). A mixture of methanol–water (pH adjusted to 3.0 with orthophosphoric acid) in the ratio of 60:40 was the mobile phase used. The analysis was performed at a flow rate of 1 ml/min isocratically and the analytes were detected at 363 nm using a photodiode array detector (Agilent, Model G1315D, USA). LC–MS analysis was carried out using system MDS SCIEX API-4000,Q-TRAP, Canada with MS/MS API-4000,Q-Trap detector with triple quadrapole mass detector. Chromatographic separation was achieved by Waters column X Terra C_18_, 250 × 4.6 mm, 5 µm and the same mobile phase used for HPLC was used (pH adjusted to 3.0 with formic acid). The ESI detection was set to positive mode. A temperature of 300 °C and scan range of 50–900 was set for the analysis. The transformed compounds were identified from the masses of products obtained in LC–MS analysis and previous reports.

### Metabolic inhibition of meloxicam

The inhibition of metabolism of meloxicam was studied using specific inhibitors of human CYP2C9 viz. clopidogrel, fenofibrate, fluoxamine and sertraline in fungal culture *C. blakesleeana* NCIM 687. The fungal culture was grown in the same medium which is used for biotransformation, and a two-stage fermentation protocol was adapted for the study. Each 20 ml of the second-stage cultures was added with 50, 100, 150, and 200 µg of inhibitors (dissolved in 100 μl DMSO) separately and incubated at 27 °C for 24 h. The CYP2C9 enzyme inhibited second-stage cultures were added with 2 mg each of meloxicam dissolved in 200 μl DMSO. Each inhibitor was studied in triplicates while running suitable controls. The contents of the flasks were extracted and analyzed using the procedure similar to that employed for meloxicam metabolism by *C. blakesleeana*. The percent of 5′-OH methyl meloxicam production in the cultures was calculated for studying the metabolic inhibition by inhibitors used.

### Statistical analysis

The experimental results were analyzed statistically (student’s t test) using Sigma Stat statistical software, version 11.0 (Jandel Corporation, Sam Rafael, CA, USA) software program package. The *P* values below 0.01 were considered statistically significant. The results were expressed as mean ± SD. The experiments were carried out in triplicates.
